# Contrast-Induced Encephalopathy After Neurointerventional Procedures: A Series of Three Cases

**DOI:** 10.1155/crnm/4384841

**Published:** 2025-09-16

**Authors:** Kaiying Wang, Rudy Goh, Shaddy El-Masri, Stephen Bacchi, Sandy Patel, Jim Jannes, Timothy Kleinig

**Affiliations:** ^1^Department of Neurology, Royal Adelaide Hospital, Adelaide 5000, South Australia, Australia; ^2^Faculty of Health and Medical Sciences, University of Adelaide, Adelaide 5005, South Australia, Australia; ^3^College of Medicine and Public Health, Flinders University, Bedford Park, Adelaide 5042, South Australia, Australia

**Keywords:** case report, computed tomography, contrast-induced encephalopathy, differential diagnosis, radiology, stroke management

## Abstract

**Introduction:** Contrast-induced encephalopathy (CIE) is a rare complication that may occur following contrast administration during endovascular interventions. The phenomenon is well-described following coronary angiography but reports following endovascular neurointerventional procedures are sparse. This study aims to describe the clinical presentation, treatment and outcome of CIE in a tertiary metropolitan hospital in South Australia.

**Methods:** This study describes a case series of 3 patients diagnosed with CIE following cerebral angiography within a 1-year period in a tertiary hospital.

**Results:** All patients developed slowly progressive (and/or new) focal or global neurological deficits 2–7 h postprocedure and exhibited characteristic neuroimaging findings. Two of three patients made an excellent recovery with supportive care, improving after 48–72 h, although one died due to the severity of her associated stroke.

**Conclusion:** CIE should be suspected in patients presenting with acute neurological deterioration following cerebral angiography. Supportive care may lead to full recovery. Multicentre prospective cohort studies are required to better define associations, diagnostic criteria and interventions to prevent and/or treat this condition.

## 1. Introduction

CIE is an uncommon cause of encephalopathy following the administration of contrast medium for medical imaging. Most described cases of CIE have been associated with intra-arterial contrast administration during either coronary or cerebral angiography [1–6]. Common clinical symptoms included focal neurological deficits, encephalopathy, speech and visual disturbances [2, 3, 5–7]. The diagnosis is often described as a diagnosis of exclusion, made clinically, and supported by imaging findings. Although CIE generally has a favourable prognosis, it may be associated with increased mortality during endovascular thrombectomy (EVT) for acute ischaemic stroke. This study aims to describe the clinical presentation, treatment and outcome of CIE in a tertiary metropolitan hospital in South Australia. It specifically focuses on cerebral angiography and highlights the varying presentations, which can be more confounding in the setting of ischaemic stroke.

## 2. Methods

### 2.1. Study Design, Setting and Population

The study involved patients who received cerebral angiography complicated by CIE from 1st January–31st December 2023 at a Comprehensive Stroke Centre in South Australia (Royal Adelaide Hospital).

### 2.2. Ascertainment of Data

Data were collected from the existing, prospectively maintained neurology and stroke registries. Data collection is performed by medical and nursing staff on an ongoing basis. Cases were ascertained on a prospective basis, and using ICD-10 codes. The diagnosis of CIE in each case was verified by a neurovascular consultant and neuroradiologist.

## 3. Results

### 3.1. Case 1

**Figure 1:**
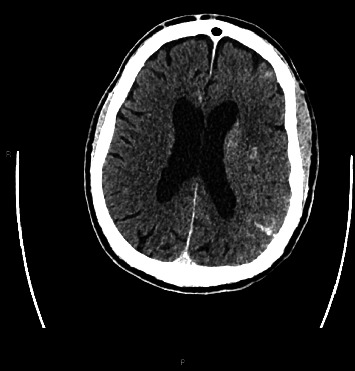
Postcontrast CT demonstrating extensive hyperdense cortical oedema with contrast enhancement and loss of grey–white matter differentiation consistent with CIE.

**Figure 2:**
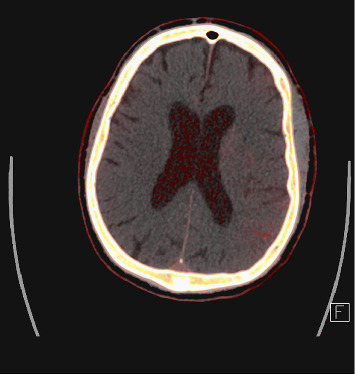
Iodine map demonstrating pooling of iodinated contrast in red.

**Figure 3:**
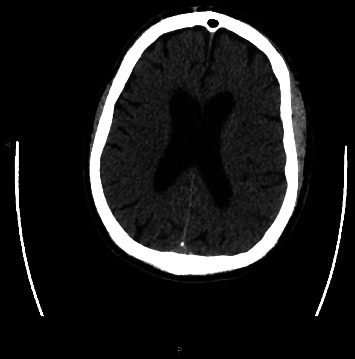
Postcontrast CT with iodine subtraction, noting the absence of contrast enhancement compared to Figure 1.

An 80-year-old man presented with a left middle cerebral artery (MCA) syndrome secondary to left MCA (M2 branch) occlusion. His last known well time was 3 h: 14 min prearrival. His initial National Institute of Health Stroke Scale (NIHSS) was 6. He underwent thrombolysis with tenecteplase and EVT. His left extracranial internal carotid artery (ICA) stenosis was stented and aspiration thrombectomy was performed with successful (thrombolysis in cerebral infarction scale 2c [TICI2c]) recanalisation. Tirofiban was commenced postprocedurally due to in-stent platelet aggregation. One hour post procedure, he had worsening left MCA syndrome with a NIHSS of 8. His observations were unremarkable, with a blood pressure of 116/56, and he was afebrile. His medications were aspirin, ticagrelor, atorvastatin, amlodipine and perindopril. CT angiography demonstrated a patent ICA stent and left M2. Repeat multimodal CT perfusion imaging performed due to further clinical deterioration 7 h later demonstrated extensive hyperdense cortical oedema with contrast enhancement and loss of grey–white matter differentiation within the left MCA territory (Figures 1, 2, 3), consistent with CIE. No treatment other than intravenous fluids was given. MRI on Day 2 showed patchy regions of infarct through the left MCA territory with no evidence of haemorrhagic transformation. He had fluctuating encephalopathy and neurological deficits for 3 days postangiography but demonstrated a consistent trend towards clinical recovery after Day 3. He was discharged to inpatient rehabilitation with residual mixed receptive and expressive dysphasia, NIHSS 1.

### 3.2. Case 2

**Figure 4:**
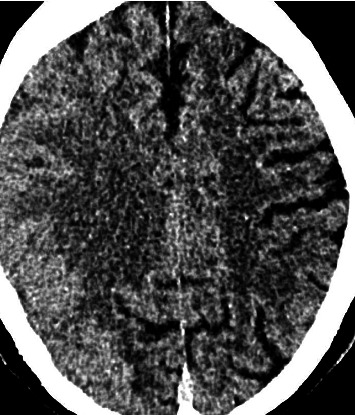
Diffuse right hemispheric cerebral oedema consistent with CIE.

A 76-year-old lady had successful insertion of a flow-diverting stent for a right ophthalmic artery aneurysm. Her procedure was complicated by an air embolus intraoperatively. She was transferred for hyperbaric treatment postoperatively after left arm weakness (NIHSS 3) was observed postprocedure. Multimodal CT imaging demonstrated patent right ICA stent. Despite supportive medical therapy, she deteriorated clinically with a complete right MCA syndrome with an approximate NIHSS 19 at 8 h postprocedure. Her blood pressure was 150/67. Her C-reactive protein was normal, and there were no electrolyte abnormalities. Her medications were aspirin, ticagrelor, escitalopram and rosuvastatin. Repeat CT perfusion imaging showed diffuse right hemispheric cerebral oedema consistent with CIE (Figure 4). Due to severe obtundation, she required intubation and ventilation overnight. She was treated with dexamethasone and levetiracetam. She was successfully extubated on Day 2 with complete resolution of neurological deficits on Day 3. MRI on Day 4 demonstrated resolution of right hemispheric cerebral oedema without infarction.

### 3.3. Case 3

**Figure 5:**
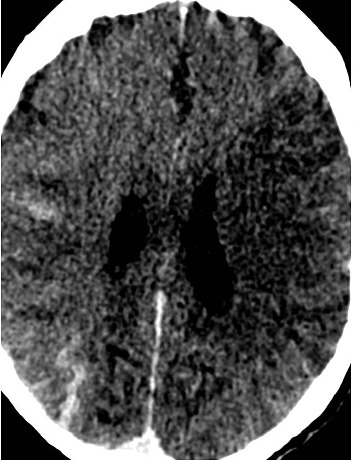
Large volume established left MC territory infarction, with widespread oedema of the subcortical white matter and contrast enhancement of subarachnoid spaces.

A 94-year-old lady presented with a complete left MCA syndrome secondary to left ICA occlusion requiring EVT. Her initial NIHSS was 24. Despite intensive suction thrombectomy, there was significant residual thrombus in her ICA resulting in TICI 2B recanalisation. As expected, she experienced clinical deterioration at 7 h postprocedure with obtundation and left-sided focal motor seizures. Her blood pressure was 150/85. Her serum electrolytes were normal. Her medications were aspirin, tirofiban, candesartan, metformin, nifedipine and pantoprazole. Repeat CT brain demonstrated a large volume established left MCA territory infarction, with widespread oedema of the subcortical white matter and contrast enhancement of the subarachnoid spaces (Figure 5). Dual energy CT imaging excluded subarachnoid haemorrhage. Given her deteriorating clinical state, extensive infarction and pre-existing wishes, life-sustained treatments were withdrawn. The patient dies after 4 days of palliative care.

## 4. Discussion

CIE can present with a constellation of neurological symptoms that can mimic stroke. CIE is hypothesised to occur due to neurotoxicity, with disturbance of blood–brain barrier and endothelial dysfunction due to hyperosmolarity of contrast agents [1]. There is a higher incidence with high osmolar contrasts and has been observed with iopromide [3, 5], ioversol [5] and iohexol [7], especially during intra-arterial injection into cranial vessels. Other risk reported factors include hypertension, renal failure and diabetes [3]. It is also notable that it can occur frequently in patients with pre-existing severe cerebral vasculopathies, such as cerebral autosomal dominant arterioropathy with subcortical infarcts and leucoencephalopthy (CADASIL). Typical radiological findings suggestive of CIE include abnormal cortical enhancement and striatal contrast enhancement [2, 3, 6, 8]. When suspected, dual energy CT may be performed to differentiate contrast from blood by subtracting the specific K edge (peak X ray absorption) of iodine [9]. CIE without corresponding radiological findings has been reported [4, 7, 10], but these limited studies were compromised by potentially incorrect CIE diagnoses as other potential alternative causes of encephalopathy were not adequately excluded.

In this case series, we diagnosed CIE following intracranial endovascular procedures for acute ischaemic stroke when there was a combination of clinical deterioration and imaging findings suggestive of CIE [3]. Iopromide was the contrast agent administered in all three cases (see Table 1). This study demonstrated that clinical deterioration occurred between 1 hour and 7 hours after contrast administration, consistent with the findings in previous studies [2, 4, 5, 7, 10]. These patients often had altered mental status on review but also had varying deficits such as motor weakness, aphasia, gaze preference and seizures [2, 4, 5, 7, 10].

An important differential diagnosis to consider in cases with CIE is hyperperfusion syndrome (HPS). HPS is a severe complication following revascularisation postthrombectomy. HPS should be considered in cases with an increase of > 100% in cerebral blood flow (CBF) from baseline after recanalisation. This can be assessed through imaging modalities such as transcranial colour duplex, CT perfusion and single-photon emission computed tomography. HPS can present with mild to severe neurological symptoms of headache, reduced conscious state, focal seizures and weakness, similar to CIE. Suggestive imaging findings include white matter oedema, particularly in the posterior parieto-occipital region. In the acute stroke setting, lowering blood pressure to < 140/80 mmHg consistently postprocedure may reduce HPS risk [11]. In the described cases, an increase in CBF was not identified in repeat CT angiography at Code Stroke, and the patients were not severely hypertensive postreperfusion. The clinical picture and investigations were therefore less likely to reflect HPS and more consistent with CIE.

CIE is associated with a favourable outcome [8], with improvement usually occurring rapidly within 48–72 h [5, 10]. Both Cases 1 and 2 had favourable outcomes by 72 h postonset of CIE. Case 1 received supportive care with intravenous fluid only, and Case 2 received additional therapy with levetiracetam and dexamethasone, as Case 2 had significant deterioration requiring intubation and Intensive Care Unit admission. Previous studies described that a majority of patients received only supportive care with intravenous fluids, with a minority receiving antiseizure medications and corticosteroids depending on clinical presentation [5, 7]. Case 3 had a poor outcome; however, this was attributable to her large established infarct volume prethrombectomy rather than CIE. This is consistent with previous studies demonstrating that other patient factors, such as poor functional baseline precluding sustained life-supportive care can contribute to death following CIE [12].

There are several limitations in this study. Firstly, this case series has a small sample size of three patients from a single centre, which limits the generalisability of these findings. Secondly, case two had associated air embolism, which may have been a confounding factor. The volume of contrast administered during angiography was not recorded in these patients. As this was a retrospective case series, selection bias cannot be excluded. The clinical findings described were dependent on clinical documentation by medical staff, which were assumed to be accurate. However, all stroke cases are prospectively and consecutively reviewed at a weekly neuroimaging and stroke aetiological classification meeting.

Future research in this area should seek to establish formal diagnostic criteria for CIE, as well as determine the association between contrast agents (such as volume and brand) and incidence of CIE. It is uncertain whether clinical deterioration with cortical dysfunction is directly modulated by local contrast extravasation, or whether CIE may induce cortical spreading depolarisation (CSD) and/or focal seizures. It is also possible that an alternative pathology, such as air embolism occurring in conjunction with contrast extravasation may be causative. The striking phenomenological overlap with other poorly understood conditions associated with hemispheric encephalopathy, and cerebral blood–brain barrier dysfunction requires further study (e.g., Stroke like Migraine Attacks after Radiotherapy [SMAR]) syndrome and neuronal intranuclear inclusion disease). A large multicentre prospective cohort study is required to more precisely predict this phenomenon and provide targeted preventative and treatment measures.

## Figures and Tables

**Table 1:** Summary of case series.

Contrast agent	Symptoms	Duration	Treatment
Iopromide	Right hemiparesis, dysphasia	Improvement in less than 1 day	IV fluids
Iopromide	Left hemiparesis, right gaze preference	Extubated Day 2, normal power Day 3	Dexamethasone, levetiracetam
Iopromide	Reduced GCS, focal motor seizures	End-of-life care	Levetiracetam, then supportive
